# Genomic analysis of the *Staphylococcus pseudintermedius* mobilome associated with antimicrobial resistance

**DOI:** 10.3389/fmicb.2025.1640322

**Published:** 2025-10-08

**Authors:** Catarina Morais, Sofia Santos Costa, Dennis Hanke, Ana Santos, Henrike Krüger-Haker, Constança Pomba, Andrea T. Feßler, Stefan Schwarz, Isabel Couto

**Affiliations:** ^1^Global Health and Tropical Medicine, GHTM, LA-REAL, Instituto de Higiene e Medicina Tropical, IHMT, Universidade NOVA de Lisboa, Lisbon, Portugal; ^2^Institute of Microbiology and Epizootics, Center for Infection Medicine, School of Veterinary Medicine, Freie Universität Berlin, Berlin, Germany; ^3^Veterinary Centre for Resistance Research (TZR), School of Veterinary Medicine, Freie Universität Berlin, Berlin, Germany; ^4^Laboratory of Antimicrobial Resistance, CIISA, Faculty of Veterinary Medicine, University of Lisbon, Lisbon, Portugal

**Keywords:** *Staphylococcus pseudintermedius*, mobilome, antimicrobial resistance, WGS, plasmids, transposons, SCC*fus*, mobile genetic elements

## Abstract

The increasing antimicrobial resistance (AMR) in *Staphylococcus pseudintermedius* causing skin and soft-tissue infections (SSTIs) in companion animals is a public health concern. The aim of this study was to verify if mobile genetic elements (MGEs), in particular plasmids, are related to the carriage of AMR genes among circulating and clinically relevant *S. pseudintermedius*. In total, 56 *S. pseudintermedius*, representing predominant and emerging clonal lineages associated with SSTIs in dogs and cats collected in Lisbon (Portugal), were subjected to plasmid DNA extraction and digestion with *Eco*RI and *Xba*I. Each unique restriction pattern was assigned to a plasmid profile. A subset of 17 strains was further selected for hybrid whole genome sequencing (WGS) on Oxford Nanopore MinION and Illumina MiSeq platforms. Thirty-one of the 56 *S. pseudintermedius* strains carried one or more plasmid(s), mostly of small or medium sizes, corresponding to eight plasmid profiles. Two of the identified plasmids carried AMR determinants; plasmid pSP-G3C4, isolated from ST71 strains, carried the tetracycline resistance gene *tet*(K) and plasmid pSP5912, isolated from a ST2061 strain, harbored the *qacG* biocide resistance gene. Other AMR determinants were detected as part of MGEs integrated into the bacterial chromosomal DNA, namely Tn*552*, Tn*552*-like, Tn*553*, Tn*916*, Tn*5405*-like, Tn*5801*, Tn*5801*-like GI*6287* and pRE25-like elements. In addition, a new chromosomal cassette, carrying *fusC*, was identified in a ST1183 strain. The 12 methicillin-resistant *S. pseudintermedius* studied carried staphylococcal cassette chromosome *mec* (SCC*mec*) type III (*n* = 5), SCC*mec* type IVg (*n* = 3), SCC*mec*_NA45_ (*n* = 1), ΨSCC*mec*_57395_ (*n* = 1), the recently described cassettes SCC*mec*_7017–61515_ (*n* = 1), or SCC*mec* type V(T)_SL/154_ (*n* = 1). Most strains carried intact prophages without AMR determinants. Intact restriction-modification systems were detected in 12 out of the 17 strains and CRISPR/Cas in five strains, four of which were methicillin-susceptible. The results of this study suggest that the AMR content in *S. pseudintermedius* is mainly related to MGEs integrated into the chromosomal DNA rather than located on plasmids. These results provide important insights that may lead to a better understanding of multidrug resistance in *S. pseudintermedius* towards improved SSTIs treatment in companion animals.

## 1 Introduction

*Staphylococcus pseudintermedius* is the most common pathogen associated with skin and soft-tissue infections (SSTIs) in companion animals (Lynch and Helbig, 2021), among which canine pyoderma is the most relevant. The recently updated guidelines for canine pyoderma treatment indicate that the first-line therapy for surface and superficial pyoderma is based on biocides or topical antimicrobials, when necessary (Loeffler et al., 2025). For systemic infections, the treatment includes clindamycin, lincomycin, amoxicillin–clavulanate, or first generation cephalosporins (first-line), fluoroquinolones, tetracyclines or trimethoprim-sulfamethoxazole (second-line) (Loeffler et al., 2025). For cats it was also recommended to apply biocides and the systemic use of amoxicillin–clavulanate, clindamycin or cefovecin (Wildermuth et al., 2006; Miller et al., 2023).

We recently characterized a collection of 155 *S. pseudintermedius* strains, obtained from SSTIs in companion animals between 2014 and 2018 in Lisbon (Portugal), regarding antimicrobial resistance (AMR) profiles and clonal lineages (Morais et al., 2023). In that earlier study, 45.2% of the strains had a multidrug resistance (MDR) profile, corresponding to resistance to at least one antimicrobial of three different classes (Sweeney et al., 2018), and about a third (31.0%) were methicillin-resistant *S. pseudintermedius* (MRSP). High rates of resistance were observed to most of the first- and second-line therapeutical antimicrobial agents, following the data from other studies (Feßler et al., 2022; Adiguzel et al., 2022; Afshar et al., 2023; Robb et al., 2024; Calabro et al., 2024). In addition, we detected strains resistant to fusidic acid and rifampicin (Morais et al., 2023). Fusidic acid is a topical antimicrobial agent approved for human and veterinary applications in Europe for the treatment of methicillin-resistant staphylococcal infections (Morris et al., 2017; Loeffler et al., 2025). Rifampicin, an ansamycin, which is part of the first-line treatment of tuberculosis in humans, is indicated for canine pyoderma caused by bacteria resistant to first-line therapy (systemic and topic) (Hillier et al., 2014; Miller et al., 2023; Loeffler et al., 2025) or by MRSP strains with a MDR phenotype (Hicks et al., 2021; Harbour et al., 2022; Loeffler et al., 2025). However, *S. pseudintermedius* rapidly develops rifampicin resistance (Kadlec et al., 2011; Hicks et al., 2021) and nowadays, this antimicrobial is considered “reserved” (Loeffler et al., 2025). Regarding the *S. pseudintermedius* clonal lineages circulating in Portugal, our previous study indicated that sequence type (ST) 71 remained the most frequent clonal lineage, associated with methicillin resistance and MDR profiles. Several new clonal lineages (ST258, ST551, ST241 and ST265) were also identified for the first time in Portugal (Morais et al., 2023).

Antimicrobial resistance (AMR) genes can be integrated into the chromosomal DNA or in mobile genetic elements (MGEs) like plasmids, bacteriophages, staphylococcal cassette chromosome (SCC) elements, and transposons. MGEs have been linked to clonal expansion and evolution of different bacteria, including *Staphylococcus aureus* (Brooks et al., 2020). *S. pseudintermedius* has an open pangenome with a significant presence of accessory genes, which generally correspond to MGEs (Brooks et al., 2020; Fàbregas et al., 2023; Grist et al., 2025). The presence of AMR genes in this species has been mainly correlated to the carriage of transposons, such as Tn*552* (*blaZ*), Tn*917* [*erm*(B)], Tn*5405*-like (*aadE*, *sat4*, *aphA3*) and Tn*916* [*tet*(M)] (Kadlec and Schwarz, 2012; Phumthanakorn et al., 2021). However, albeit in lower frequency, some AMR genes were also found on plasmids, like pSTS2 carrying the *tet*(K) gene or plasmids pSCS1, pSCS11 (Greene and Schwarz, 1992) and pSCS20-23 carrying the *cat* gene (Schwarz et al., 1995). While phages represent one of the most relevant mechanisms for DNA transfer in *S. pseudintermedius* (Brooks et al., 2020), AMR or virulence genes are not frequently found in these MGEs. In *S. aureus*, phages usually also do not carry AMR genes (Haaber et al., 2017), but they allow the mobility of pathogenicity islands and plasmids carrying AMR genes by transduction (Malachowa and DeLeo, 2010).

In the current study, we aimed at further analyzing representative strains from the Lisbon collection (Morais et al., 2023) and, through whole genome sequencing (WGS), obtaining information about the role of the staphylococcal mobilome in the carriage of AMR among circulating and clinically relevant *S. pseudintermedius* lineages.

## 2 Material and methods

### 2.1 Bacterial collection

The study collection comprised 56 *S. pseudintermedius* strains obtained from SSTIs in companion animals (53 dogs and 3 cats). These 56 strains, described in Supplementary Table 1, were selected from the collection of 155 *S. pseudintermedius* previously characterized (Morais et al., 2023), according to the following criteria: (i) all strains from ST71 and ST157, which correspond to the two most frequent STs in the collection; (ii) strains from relevant STs in the European context (ST45, ST118, ST241, ST258, ST265 and ST551); (iii) strains with phenotypes of interest, namely resistance to fusidic acid, tetracycline or rifampicin.

### 2.2 Plasmid DNA profiling

Plasmid DNA (pDNA) was extracted with the NZYMiniprep kit (NZYtech, Portugal) or QIAGEN Plasmid Midi Kit (Qiagen, Germany), adding 35 μg/mL of lysostaphin (Sigma-Aldrich, Missouri, USA) in the cell lysis step, followed by incubation at 37 *^o^*C for 90–120 min. Plasmids were classified according to their migration in the gel before and after digestion with *Xba*I and *Eco*RI restriction enzymes (NZYtech), as small (≤ 5 kb), medium (> 5 kb and < 23 kb), or large (≥ 23 kb) plasmids, using the weight markers Lambda DNA/*Hin*dIII Ladder and GeneRuler 1 kb DNA Ladder (Thermo Fisher Scientific, Waltham, USA). Each unique restriction pattern was assigned to a plasmid profile, later confirmed by WGS data.

### 2.3 Genomic DNA extraction and whole genome sequencing (WGS)

A subset of 17 strains out of the 56 representative *S. pseudintermedius* was selected for WGS analysis by a hybrid approach with Oxford Nanopore and Illumina technologies, generating long-reads and short paired-end reads, respectively (Supplementary Table 1). These 17 strains were selected by the following criteria (i) at least one strain from each plasmid profile; (ii) at least one strain per lineage; (iii) all fusidic acid and rifampicin resistant strains, excluding one rifampicin strain (BIOS-V241) sharing the plasmid profile and lineage of BIOS-V240 (sequenced); (iv) strains from the predominant lineages without plasmids.

Genomic DNA was obtained from 1 mL of overnight culture (Tryptic Soy Broth at 37 *^o^*C) using the MagAttract HMW DNA Kit (Qiagen) following the manufacturer’s protocol. The Native Barcoding Kit-24 (SQK-NBD112-24, Oxford Nanopore Technologies, Oxford, UK) was used to prepare the sequencing libraries with 400 ng of DNA for MinION. The barcoded libraries were pooled, to carry out multiplexed sequencing, and loaded onto a MinION FLO-MIN106 flow cell v9.4.1 and sequenced in a MinION Mk1C. For Illumina, the libraries were prepared using the Nextera XT DNA Library Preparation Kit (Illumina, Inc., San Diego, USA) according to the manufacturer’s recommendations. The 2 × 300-bp paired-end sequencing in 40-fold multiplexes was performed on the Illumina MiSeq platform with the MiSeq Reagent Kit v3 (Illumina). DNA quantification was carried out using the Qubit*™* 4 fluorometer (Invitrogen, NY, USA) with the Qubit*™* dsDNA HS assay kit (Invitrogen). Base-calling and demultiplexing of MinION read files were conducted via MinKNOW v23.04.5 and Porechop v0.2.4, respectively. The quality of the long-reads was assessed in LongQC v1.2.0c (Fukasawa et al., 2020) and short fragments with low quality were removed with Filtlong v0.2.1. Short-reads were trimmed with TrimGalore (RRID:SCR_011847) v0.6.10 and their quality assessed through FastQC v0.12.0^1^.

### 2.4 Genome assembly and annotation

Genomes were *de novo* assembled with Flye v2.9.3 (Kolmogorov et al., 2019) and polished with NextPolish v1.4.1 (Hu et al., 2020). The results obtained from Flye were compared in Geneious Prime v8.1.9 (Biomatters, Ltd., Auckland, New Zealand) to *de novo* assemblies performed with Unicycler v0.4.9 (Wick et al., 2017) and MaSuRCA v4.1.0 (Zimin et al., 2017). Annotation was accomplished with Bakta v1.8.2 (Schwengers et al., 2021). The genome completeness was analyzed with the Benchmarking Universal Single-Copy Orthologs (BUSCO) tool (Manni et al., 2021). Extra-chromosomal contigs assembled with Flye were identified as reflecting possible plasmids according to the size, the presence of a *rep* gene, coverage and circularity in Bandage v0.8.1 (Wick et al., 2015). BLASTn NCBI (Camacho et al., 2009) was used to determine the homology to other plasmids as previously described.

### 2.5 Antimicrobial resistance (AMR) genes detection

*In silico* screening for the presence of acquired AMR genes and point mutations was performed through the Comprehensive Antibiotic Resistance Database (CARD) (Jia et al., 2017; Alcock et al., 2023) and ResFinder v4.5.0 (Camacho et al., 2009; Bortolaia et al., 2020). The presence of point mutations in *fusA* and *rpoB* genes was confirmed by aligning the sequences with the genes of known susceptible *S. pseudintermedius* strains: HKU10-03 (accession no.: NC_014925.1) and FDAARGOS_930 (accession no: NZ_CP065635), also deposited at DSMZ repository as *S. pseudintermedius* strain DSM21284^T^.

### 2.6 Mobile genetic elements identification

Mobile genetic elements were identified using bioinformatics tools available online. Plasmids were predicted with PlasmidFinder v2.1 (Camacho et al., 2009; Carattoli et al., 2014), transposons and insertion sequences with MobileElementFinder v1.0.3 (Johansson et al., 2021), both available at the Center for Genomic Epidemiology^2^. PHIGARO (Starikova et al., 2020) was used to determine the content of prophages in the studied genomes and PHASTEST v3.0 web server (Zhou et al., 2011; Arndt et al., 2016) to identify and classify them as intact, questionable or incomplete prophages. All identified prophages were BLASTn searched against the NCBI Virus database. Staphylococcal cassette chromosome *mec* (SCC*mec*) was first screened with SCC*mec*Finder 1.2^3^, which is available for *S. aureus*. The results obtained were then compared with the whole genome sequence of the strain, identifying putative integration site sequences (ISSs) for SCC*mec* as described previously for *S. aureus* (Ito et al., 2004) and *S. pseudintermedius* (Perreten et al., 2013). A BLASTn search was performed on the resulting DNA sequence to identify the most similar SCC*mec* type described for *S. pseudintermedius.* These putative ISSs were also used to delimit SCC*fus*.

### 2.7 Identification of restriction-modification systems and clustered regularly interspaced short palindromic repeats

Restriction-modification (R-M) systems were predicted using the information provided by the rmsFinder tool (Roberts et al., 2015) and REBASE database^4^. Clustered Regularly Interspaced Short Palindromic Repeats (CRISPR) sequences and Cas proteins were detected with CRISPRCasFinder^5^ (Couvin et al., 2018), using the software default parameters. The classification provided by the software was compared with the classification suggested by Rossi et al. (2019). Only the complete systems, containing both CRISPR and Cas proteins, were considered for analysis.

## 3 Results

### 3.1 Plasmid profiling

A first set of 56 representative *S. pseudintermedius* strains was selected for analysis of plasmid content. Plasmid DNA extraction revealed the presence of plasmids in 31 out of the 56 strains screened, mostly of small or medium size. These corresponded to eight distinct plasmid profiles (P1–P8) (Table 1). Plasmid profile P1 was the most frequently detected (16/31). Plasmid profile P2 included two different plasmids with indistinguishable restriction profiles, size and main features that were carried by strains from different STs. We also found strains from the same ST carrying different plasmids as well as the same plasmid carried by strains from different lineages (pBIOS236). No plasmids were detected in strains from lineages ST25, ST45, ST157, ST265, ST422, ST497, ST924, ST2055, ST2059, ST2099 and ST2102 (Table 1).

**TABLE 1:** Plasmid profiles determined among the 56 representative *S. pseudintermedius* strains, detailed through whole genome sequencing (WGS) analysis.

Plasmid profile	ST^(1)^	No. strains/profile	Strains studied by WGS	Plasmid	Plasmid size (bp)	AMR genes detected on plasmids	BLAST result (best hit)		
							Accession number	% identity	% query cover
**P1**	71	16	**BIOS-V104**	**pSP-G3C4**	4,439	*tet*(K)	MN612109.1	100	100
			**BIOS-V144**	**pSP-G3C4**			MN612109.1	100	100
			**BIOS-V299**	**pSP-G3C4**			MN612109.1	99.96	100
**P2^(2)^**	258	3	**BIOS-V141**	**pCUVET18-79.2**	3,043	None	CP119700.1	99.56	100
	551		**BIOS-V227**	**pCUVET16-803.2**	3,043	None	CP119697.1	99.96	100
**P3**	1183	1	**BIOS-V212**	**pBIOS212**	3,660	None	CP129356.1	99.86	100
**P4**	241	1	**BIOS-V236**	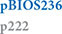	15,280 15,203	None None	CP132401.1^(3)^ CP011490.1	96.01 99.85	100 98.0
**P5**	241	6	**BIOS-V218**	**p222**	15,203	None	CP011490.1	99.85	98.0
**P6**	2061	2	**BIOS-V240**	**pSP5912**	2,743	*qacG*	CP009121.1	100	100
**P7**	118	1	**BIOS-V262**	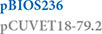	15,281 3,043	None None	CP132401.1^(3)^ CP119700.1	95.99 99.85	100 100
**P8**	2109	1	**BIOS-V259**	**pBIOS259**	2,469	None	CP119718.1	83.44	100
**No plasmid**	25; 45; 71; 157; 265; 422; 497; 924; 2055; 2059; 2099; 2102	25	**BIOS-V16, V64, V127, V179, V237, V292**	–	–	–	–	–	–

ST, sequence-type; AMR, antimicrobial resistance. 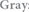: plasmid name assigned based on >95% identity to sequences in NCBI; 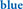: plasmid designation newly assigned in this work because <95% similarity to sequences in NCBI; 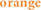: plasmid already published in NCBI as “unnamed”; we now propose a specific name based on the strain number. ^(1)^ Morais et al., 2023. Front. Microbiol. 14:1167834. doi: 10.3389/fmicb.2023.1167834. ^(2)^The restriction profile observed was identical, along with the size and the main features of the plasmids. ^(3)^The 29,587-bp plasmid UVET16-496.1 (accession number CP132401.1) corresponds to a duplication of a ≈ 15,000-bp region, which presents >95% identity with pBIOS236.

### 3.2 Genomic characterization of *S. pseudintermedius* by WGS

**FIGURE 1:**
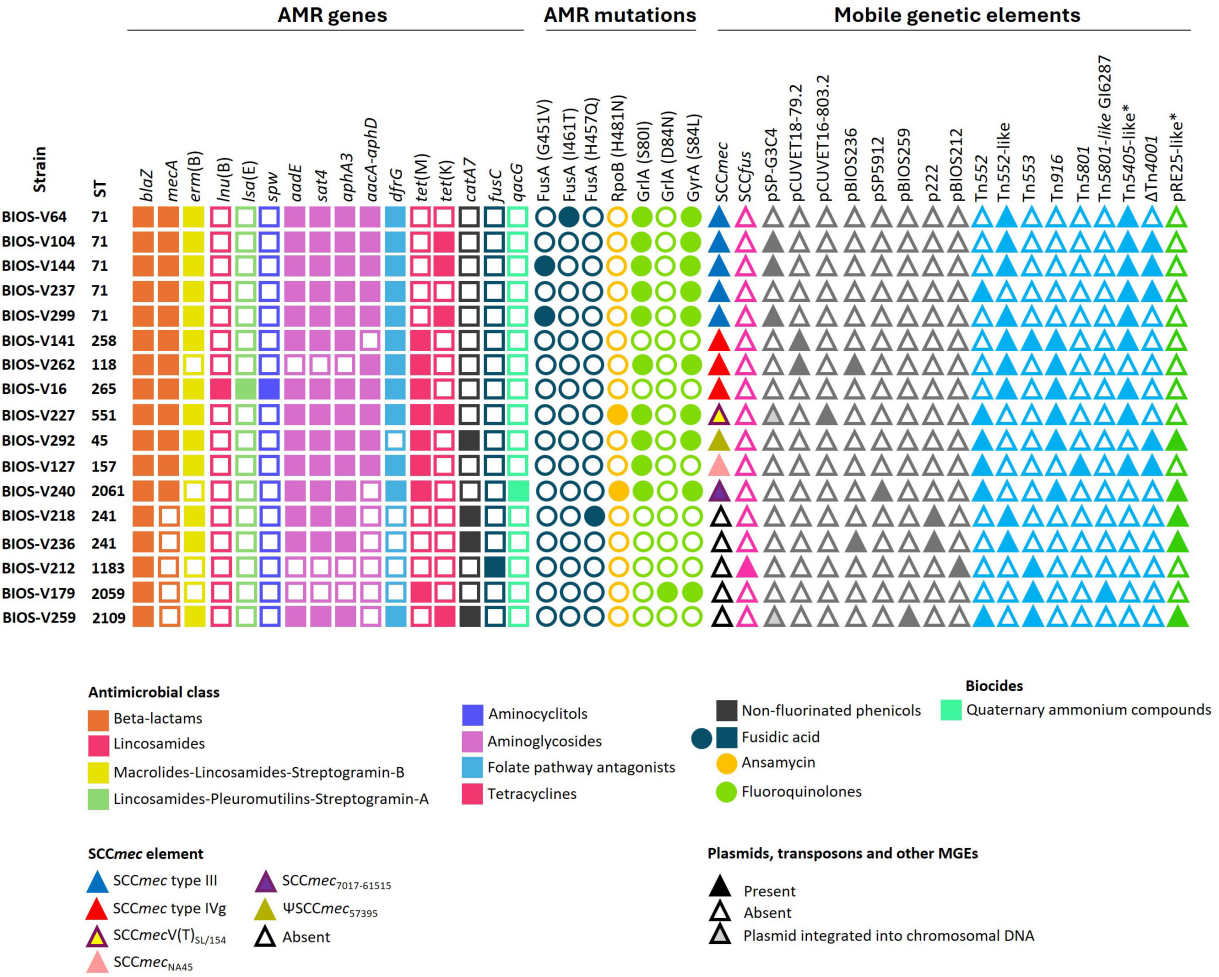
Clonal lineage, antimicrobial resistance (AMR) determinants and mobile genetic elements (MGEs) of the 12 MRSP and 5 MSSP strains selected for whole genome sequencing. Squares and circles correspond to AMR genes and point mutations, respectively. Triangles correspond to MGEs. *Include variants of this element carrying different AMR genes (see Table 3).

A subset of 17 strains, selected according to plasmid profile and clonal lineage and including 12 MRSP strains and five methicillin-susceptible (MSSP) strains, was further characterized by hybrid WGS (Figure 1).

The *de novo* assembly of the 17 genomes resulted in closed circular chromosomes (99.3%–99.6% completeness), with a GC content of about 37.5%. The size of the genomes varied between 2.5 and 2.9 Mbp, with 0–2 plasmids. Interestingly, the GC content of all plasmids identified in this work was lower, ranging between 28.1 and 33.4%. Detailed information on the WGS data is presented in Table 2.

**TABLE 2:** Characteristics of the 17 *S. pseudintermedius* strains and respective genomes studied by whole genome sequencing.

Strain	V64	V104	V144	V237	V299	V141	V262	V16	V227	V292	V127	V240	V259	V218	V236	V212	V179
Source	Dog	Dog	Dog	Dog	Dog	Dog	Dog	Dog	Dog	Cat	Dog	Cat	Dog	Dog	Dog	Dog	Dog
ST	71	71	71	71	71	258	118	265	551	45	157	2061	2109	241	241	1183	2059
Completeness (%)	99.6	99.6	99.6	99.6	99.6	99.6	99.3	99.6	99.3	99.6	99.6	99.6	99.6	99.6	99.6	99.6	99.3
**Genome size (bp)**																	
C	2,767,512	2,892,838	2,727,322	2,923,202	2,837,639	2,726,435	2,672,517	2,734,611	2,713,958	2,592,552	2,675,443	2,663,916	2,717,109	2,618,153	2,600,244	2,562,710	2,539,371
P	-	4,439	4,439	–	4,439	3,043	3,043 15,281	–	3,043	–	–	2,743	2,469	15,203	15,203 15,280	3,660	–
**GC content (%)**																	
C	37.5	37.4	37.5	37.4	37.5	37.6	37.6	37.6	37.4	37.6	37.5	37.5	37.5	37.7	37.6	37.6	37.7
P	–	30.1	30.1	–	30.1	29.5	29.5 33.0	–	29.6	–	–	30.2	33.4	28.1	33.0 28.1	29.8	–
**Other features**																	
CDS	2,611	2,805	2,552	2,874	2,751	2,576	2,518	2,599	2,528	2,375	2,486	2,518	2,570	2,469	2,427	2,344	2,307
tRNA	59	59	59	59	59	59	59	59	59	59	59	59	59	59	59	59	59
tmRNA	1	1	1	1	1	1	1	1	1	1	1	1	1	1	1	1	1
rRNA	19	19	19	19	19	19	19	19	19	19	19	19	20	19	19	19	19
ncRNAs	17	20	19	17	18	19	20	23	19	20	23	20	17	19	17	18	21
ncRNA regions	28	26	26	26	26	28	28	28	28	28	25	28	28	26	28	26	28
**GenBank accession number**																	
C	CP193748	CP193746	CP193741	CP193730	CP193720	CP193743	CP193723	CP193749	CP193734	CP193722	CP193745	CP193728	CP193726	CP193736	CP193731	CP193738	CP193740
P	–	CP193747	CP193742	–	CP193721	CP193744	CP193724 CP193725	–	CP193735	–	–	CP193729	CP193727	CP193737	CP193732 CP193733	CP193739	–

ST, sequence-type; GC, guanine-cytosine; C, chromosome; P, plasmid; CDS, coding sequence.

#### 3.2.1 Identification of AMR determinants

In the previous study, the strain collection was characterized regarding antimicrobial susceptibility phenotypes by disk diffusion and PCR screening of several AMR genes (Morais et al., 2023; Supplementary Table 1). WGS analysis allowed the identification of additional resistance genes in the chromosomal DNA of some strains, namely *aadE*, *sat4*, *lsa*(E), and *lnu*(B) (Figure 1). The *fosB* gene, related to fosfomycin resistance in *S. aureus* (Fu et al., 2016) was present in all 17 strains. Mutations in the quinolone resistance determining regions (QRDR) of the target genes *grlA* and *gyrA*, and corresponding amino acid exchanges, were previously identified (Morais et al., 2023) and now confirmed through WGS. Four of the five strains resistant to fusidic acid carried mutations in the *fusA* gene that resulted in the amino acid exchanges G451V, H457Q or I461T in FusA. The remaining strain carried the *fusC* gene. Rifampicin resistance was associated with a mutation in the *rpoB* gene that resulted in the amino acid exchange H481N in RpoB of the two resistant strains sequenced (BIOS-V227 and BIOS-V240) (Figure 1). Regarding tetracycline resistance, the determinants previously detected, *tet*(M) and *tet*(K) (Morais et al., 2023), were now found located either in the chromosomal DNA [*tet*(M)] or on free or integrated plasmids [*tet*(K)], in different combinations, as detailed below.

#### 3.2.2 Mobile genetic elements and AMR genes

Tables 1, 3–5, and Figure 1 detail the distribution of AMR genes and MGEs identified in the genomes of the 17 sequenced strains.

**TABLE 3:** Antimicrobial resistance (AMR) genes carried by mobile genetic elements and barriers to horizontal gene transfer (HGT) identified in *S. pseudintermedius* strains.

Strain	V64	V104	V144	V237	V299	V141	V262	V16	V227	V292	V127	V240	V259	V218	V236	V212	V179
ST	71	71	71	71	71	258	118	265	551	45	157	2061	2109	241	241	1183	2059
**Plasmids**																	
Plasmid(s)	–	pSP-G3C4	pSP-G3C4	–	pSP-G3C4	pCUVET18-79.2	pCUVET 18-79.2 pBIOS236	–	pCUVET 16-803.2	–	–	pSP5912	pBIOS259	p222	p222 pBIOS236	pBIOS212	–
Plasmid AMR genes	–	*tet*(K)	*tet*(K)	–	*tet*(K)	–	–	–	–	–	–	*qacG*	–	–	–	–	–
*mec*																	
SCC*mec*^1^	III	III	III	III	III	IVg	IVg	IVg	V(T)_SL/154_	ΨSCC*mec*_57395_	NA45	7017-6151	–	–		–	–
Heavy metal R genes^2^	–	–	–	–	–	–	–	–	*cadA*	*arsB*, *arsC*, *arsR*, *cadA*, *cadD*, *copA*	*arsB*, *arsC, arsR, copA*	–	–	–	–	–	–
**Transposons**																	
Tn*552*	–	–	–	*blaZ*	–	–	–	–	*blaZ*	*blaZ*	*blaZ*	*blaZ*	*blaZ*	–	–	–	–
Tn*552*-like	*blaZ*	*blaZ*	*blaZ*	–	*blaZ*	*blaZ*	*blaZ*	*blaZ*	–	–	–	–	–	*blaZ*	*blaZ*	–	–
Tn*553*	–	–	–	–	–	*blaZ*	–	–	–	–	–	–	*blaZ*	–	–	*blaZ*	*blaZ*
Tn*916*	–	–	–	–	–	*tet*(M)	*tet*(M)	*tet*(M)	*tet*(M)	*tet*(M)	–	*tet*(M)	–	–	–	–	–
Tn*5801*	–	–	–	–	–	–	–	–	–	–	*tet*(M)	–	–	–	–	–	–
Tn*5801*-like GI*6287*	–	–	–	–	–	–	–	–	–	–	–	–	–	–	–	–	*tet*(M)
Tn*5405*-like (Variant 1)	–	*aadE, aphA3* *sat4,erm*(B), *dfrG*	*aadE, aphA3* *sat4,erm*(B), *dfrG*	*aadE, aphA3* *sat4,erm*(B), *dfrG*	*aadE, aphA3* *sat4,erm*(B), *dfrG*	–	–	–	*aadE, aphA3* *sat4,erm*(B), *dfrG*	–	–	–	–	–	–	–	–
Tn*5405*-like (Variant 2)	–	–	–	–	–	*aadE, aphA3* *sat4*, *erm*(B)	–	–	–	–	–	–	–	–	–	–	–
Tn*5405*-like (Variant 3)	*aadE, aphA3* *sat4,erm*(B), *dfrG*	–	–	–	–	–	–	–	–	–	*aadE, aphA3* *sat4,erm*(B), *dfrG*	–	–	–	–	–	–
Tn*5405*-like (Variant 4)	–	–	–	–	–	–	–	*aadE, aphA3*, Δ*sat4,erm*(B), *aadE, lnu*(B), *lsa*(E), *spw*	–	–	–	–	–	–	–	–	–
ΔTn*4001*	–	*aacA-aphD*	*aacA-aphD*	*aacA-aphD*	–	–	–	–	–	*aacA-aphD*	*aacA-aphD*	–	–	–	–	–	–
**Other elements**																	
pSP-G3C4-like	–	–	–	–	–	–	–	–	*tet*(K)	–	–	–	*tet*(K)	–	–	–	–
pRE25-like	–	–	–	–	–	–	–	–	–	*aadE, aphA3, sat4, erm*(B), *catA7*	–	*aadE,aphA3, sat4*	*aadE,aphA3, sat4,erm*(B), *catA7*	*aadE, aphA3, sat4, erm*(B), *catA7*	*aadE, aphA3, sat4, erm*(B), *catA7*	–	–
SCC*fus*	–	–	–	–	–	–	–	–	–	–	–	–	–	–	–	*fusC*	–
**Prophages**																	
No. intact prophages	2	5	1	3	3	1	1	3	0	0	1	0	3	2	2	0	0
**Barriers to HGT**																	
R-M type	I, I	I	I, I	I, I	I, I	I	Δ I	I	II, III/IIG	II	I	–	III/IIG	Δ I	Δ I	I	–
CRISPR/Cas type	–	–	–	–	–	–	–	–	IIIA, IIIA, IIC	–	–	–	–	IIIA	IIIA	IIC	IIC

ST, sequence-type; SCC, staphylococcal cassette chromosome; R-M, restriction-modification system; CRISPR/Cas, Clustered Regularly Interspaced Short Palindromic Repeats. ^1^ SCC*mec* type III (*mec* gene complex A; *ccrA3*/*ccrB3*); SCC*mec* type IVg (*mec* gene complex B; *ccrA2*/*ccrB2*); SCC*mec* type V(T)_SL/154_ (*mec* gene complex C2; *ccrA1*/*ccrB6*); SCC*mec*_NA45_ (*mec* gene complex C1; *ccrC6*); SCC*mec*_7017–61515_ (*mec* gene complex A; *ccrC1*); ΨSCC*mec*_57395_ (*mec* gene complex C1; no *ccr* genes); ^2^ Heavy metal resistance genes carried in SCC*mec*; *arsB*, *arsC, arsR* – arsenic resistance; *copA* – copper resistance; *cadA*, *cadD* – cadmium resistance.; Δ – truncated; Tn*5405*-like variants are flanked by: variant 1: IS*1182* + ΔIS*1182* + *dfrG;* variant 2: IS*1182* + ΔIS*1182*; variant 3: ΔIS*1182* + ΔIS*1182;* variant 4: IS*1182.*

**TABLE 4:** Description of the SCC*fus* element identified in *S. pseudintermedius* strain BIOS-V212 (lineage ST1183).

Gene	Description	Orientation	Start	End	BLAST	
					BLAST results (best hit)	Accession number
*orfX*	23S rRNA methyltransferase	Sense	1	480	99.79% ID with *rlmH* (*orfX*) from *S. pseudintermedius*	CP083195.2 [32,319: 32,798]
DR_SCC	Direct repeat	Sense	463	480	–	–
	Type I site-specific R-M system, R (restriction) subunit	Sense	586	729	88.89% ID with Type I site-specific R-M system, R (restriction) subunit from *S. hominis*	CP118825.1 [117,775:117,918]
	Restriction endonuclease subunit S	Sense	722	1,948	94.68% ID with restriction endonuclease subunit S from *S. aureus*	CP049454.1 [34,667:35,893]
	PTS maltose transporter subunit IIBC	Antisense	2,107	3,549	98.13% ID with sucrose-specific PTS transporter subunit IIBC from *S. hominis*	CP031277.1 [527,329:528,771]
	Hypothetical protein	Antisense	3,799	4,020	93.64% ID (99% QC) with hypothetical protein from *S. aureus*	CP170579.1 [340,561:340,782]
	DUF1643 domain-containing protein	Antisense	4,035	4,538	99.60% ID with DUF1643 from *S. hominis*	CP142855.1 [35,204:35,707]
	DUF960 domain-containing protein	Antisense	4,554	4,865	96.14% ID with DUF960 from *S. epidermidis*	CP101316.1[1,959,091:1,959,402]
	Hypothetical protein	Antisense	4,867	4,956	100% ID with a region non-annotated from *S. hominis*	CP094724.1 [52,039:52,128]
	DUF950	Antisense	4,958	5,299	97.95% ID with truncated SAUGI family uracil-DNA glycosylase inhibitor (DUF950) from *S. hominis*	CP094724 [52,130: 52,467]
*ccrB4*	Cassette chromosome recombinase B, type 4	Antisense	5,819	7,444	95.63% ID with cassette chromosome recombinase B from *S. lugdunensis*	AP021848.1 [104,504:106,129]
*ccrA4*	Cassette chromosome recombinase A, type 4	Antisense	7,441	8,802	94.13% ID with recombinase family protein from *S. hominis*	CP054550.1 [832,438:833,799]
DUF927	DUF927 domain-containing protein	Antisense	8,989	10,761	93.97% ID with DUF927 from *S. aureus*	CP076359.1 [1,963,362:1,965,134]
	Putative *cch*-associated protein	Antisense	10,761	11,051	99.66% ID with putative *cch*-associated protein from *S. aureus* RUH-32 SCC*mec*-SCC*fus*	MK991791.1 [17,619:17,909]
	Hypothetical protein	Sense	11,222	12,292	100% ID with hypothetical protein from *S. aureus* RUH-32 SCC*mec*-SCC*fus*	MK991791.1 [18,080:19,150]
	DEAD/DEAH box helicase domain protein	Sense	12,440	14,326	99.95% ID with DEAD/DEAH box helicase domain protein from *S. aureus* RUH-32 SCC*mec*-SCC*fus*	MK991791.1 [19,244:21,184]
	Hypothetical protein	Sense	14,421	14,891	100% ID with hypothetical protein from *S. pseudintermedius*	CP076465 [2,488,518:2,488,826]
*fusC*	Fusidic acid resistance protein C	Sense	15,477	16,115	100% ID with fusidic acid resistance EF-G-binding protein FusC from *S. pseudintermedius*	CP076465 [2,487,294:2,487,932]
DR_SCC	Direct repeat		16,191	16,208	–	–

SCC, staphylococcal cassette chromosome; ID, identity; QC, query cover.

**TABLE 5:** Antimicrobial resistance (AMR) genes found in *S. pseudintermedius* mobile genetic elements (MGEs). All these MGEs were detected integrated into chromosomal DNA, except the plasmid pSP-G3C4.

AMR gene	Mobile genetic element
***blaZ***	Tn*552*, Tn*552*-like, Tn*553*
***mecA***	SCC*mec* III, SCC*mec* IVg, SCC*mec* V(T)_SL/154_, ΨSCC*mec*_57395_, SCC*mec*_NA45_, SCC*mec*_7017–6151_
***fus*C**	SCC*fus*
***tet*(K)**	pSP-G3C4, pSP-G3C4-like
***tet*(M)**	Tn*916*, Tn*5801*, Tn*5801*-like GI*6287*
***erm*(B)**	Tn*5405*-like, pRE25-like
***dfrG***	Tn*5405*-like
***catA7***	pRE25-like
***aadE***	Tn*5405*-like, pRE25-like
***aphA3***	Tn*5405*-like, pRE25-like
***sat4***	Tn*5405*-like, pRE25-like
***aacA-aphD***	ΔTn*4001*

SCC, staphylococcal cassette chromosome.

##### 3.2.2.1 Plasmids

Eleven out of the 17 sequenced strains carried eight different plasmids: pSP-G3C4, pCUVET18-79.2, pCUVET16-803.2, pSP5912, pBIOS212, p222, and the newly described pBIOS236 and pBIOS259. The sizes of these plasmids varied between 2,469 and 15,281 bp. Nine strains carried a single plasmid, and the remaining two (BIOS-V236 and BIOS-V262), had two plasmids (Table 1).

Regarding carriage of AMR genes, the 4,439-bp plasmid pSP-G3C4 shares 100% identity with a *tet*(K)-harboring plasmid from *S. pseudintermedius* strain G3C4, responsible for tetracycline resistance through increased efflux activity. The 2,743-bp *S. pseudintermedius* plasmid pSP5912 carried the *qacG* gene, associated with the efflux of quaternary ammonium compounds.

The 15,203-bp plasmid p222, carried by strains BIOS-V218 and BIOS-V236, has 99.85% identity with *S. pseudintermedius* plasmid p222 and includes the gene for the virulence factor bacteriocin BacSp222 (Wladyka et al., 2015).

The plasmid pBIOS236 was detected in BIOS-V236 (15,280 bp) and BIOS-V262 (15,281 bp). This plasmid shares 95.99–96.01% identity with a nearly 15,000-bp region that is duplicated in the 29,587-bp plasmid UVET16-496.1 and harbors *tra* genes associated with plasmid conjugation.

The 3,043-bp plasmid pCUVET18-79.2, detected in BIOS-V141 and BIOS-V262, is highly similar to the *S. pseudintermedius* plasmid pCUVET16-803.2 present in BIOS-V227 (Table 1) and to other plasmids deposited in GenBank. These plasmids carry coding sequences (CDS) for hypothetical proteins, a replication protein and CopG, which is involved in plasmid copy number control.

The 3,660-bp plasmid pBIOS212 shows 99.86% identity to *S. pseudintermedius* strain Dog009_2 plasmid unnamed1, carrying genes for a replication protein and hypothetical proteins.

Plasmid pBIOS259 is described in this study for the first time. This 2,469-bp plasmid shares 83.44% nucleotide sequence identity with plasmid pCUVET18-1255.1 previously isolated in a *S. pseudintermedius* strain and contains two CDSs for a replication protein and a hypothetical protein.

##### 3.2.2.2 Staphylococcal cassette chromosome *mec*

The *mecA* gene is carried in SCC*mec* elements, classified according to the type of the *ccr* gene complex and the class of the *mec* gene complex (International Working Group on The Classification of Staphylococcal Cassette Chromosome Elements [IWG-SCC], 2009). The 17 *S. pseudintermedius* studied included 12 MRSP strains that carried SCC*mec* type III (*n* = 5), SCC*mec* type IVg (*n* = 3), SCC*mec*_NA45_ (*n* = 1), ΨSCC*mec*_57395_ (*n* = 1), SCC*mec*_7017–61515_ (*n* = 1), and SCC*mec* type V(T)_SL/154_ (*n* = 1) (Figure 1 and Table 3). SCC*mec* type III was carried by ST71 strains, while SCC*mec* type IVg was found among strains of lineages ST265 and ST118 (both from CC227) and ST258 (a double locus variant of the previous ones, assigned to CC258).

##### 3.2.2.3 A novel staphylococcal cassette chromosome harboring *fusC* in *S. pseudintermedius*

**FIGURE 2:**
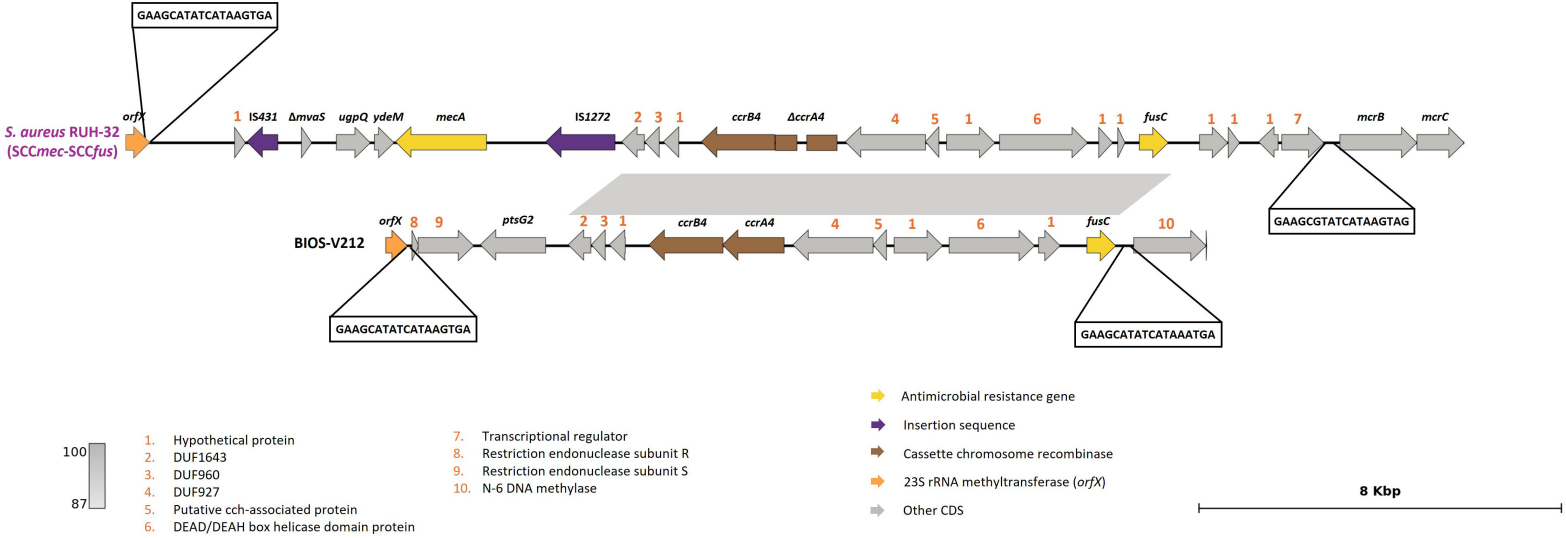
Presentation of the new SCC*fus* described in *S. pseudintermedius* carrying the *fusC* gene. Comparison with the SCC*mec*-SCC*fus* described in *S. aureus* isolate RUH-32 (MK991791). Homology is indicated through a color scale of gray: dark gray (100% homology) to light gray (87% homology). Fusidic acid resistance (*fusC*) and methicillin-resistance (*mecA*) genes are represented in yellow; cassette chromosome recombinase genes represented in brown (*ccrA4*, *ccrB4*); *orfX* in orange; insertion sequences in purple. Genes colored in gray represent other genes. The figure was generated using Genofig v1.1.

BIOS-V212 expressed resistance to fusidic acid due to the carriage of the *fusC* gene, which was located in a novel SCC element (Table 4). This 15,746-bp cassette was delimited using the putative ISS described for the assignment of SCC*mec* and carried *ccrA4* and *ccrB4* genes. This new element shared overall 99.72% identity (74% query coverage) with the previous SCC*mec*-SCC*fus* element identified in a methicillin-resistant *S. aureus* (MRSA) (Senok et al., 2019; Figure 2) yet it lacks the *orfX*-IS*1272* region containing the *mecA* gene and the region downstream of the *fusC* gene.

##### 3.2.2.4 Transposons

Most of the AMR genes found in the 17 strains sequenced were located on transposons integrated into the chromosomal DNA (Figure 1 and Tables 3, 5).

All strains carried the *blaZ* gene, located either on a Tn*552* (six strains), a Tn*552*-like element (nine strains) or a Tn*553* element (four strains, two of which also carried an additional copy of *blaZ* in Tn*552* or Tn*552*-like elements) (Table 3). The Tn*552* elements correspond to the one identified in *S. aureus* (accession no: X52734), which is delimited by two inverted repeats (Tn*552*), or a related element where a reverse transcriptase gene is inserted into one of the inverted repeats (Tn*552*-like).

**FIGURE 3:**
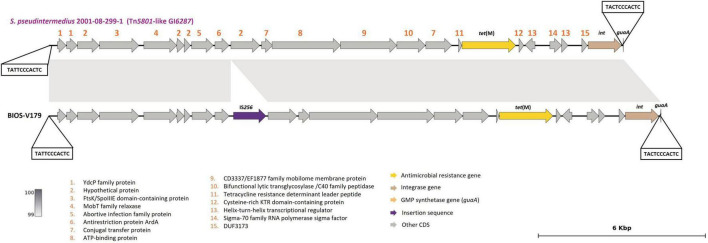
Presentation of Tn*5801*-like GI*6287* (BIOS-V179) carrying the *tet*(M) gene and comparison with strain *S. pseudintermedius* 2001-08-299-1 contig_3 (accession number: NZ_JTKO01000003.1, [contig 3, 177,382:198,007]). Minimum homology of 99% was detected between sequences (light gray color). Tetracycline resistance gene [*tet*(M)] is represented in yellow; integrase gene (*int*) is represented in brown; GMP synthetase gene (*guaA*) in orange; insertion sequence in purple. Genes colored in gray represent other genes. The figure was generated using Genofig v1.1.

The *tet*(M) gene, associated with tetracycline resistance, was found in eight strains, located either on a Tn*916* element (*n* = 6), Tn*5801* (*n* = 1) or Tn*5801*-like Genomic Island 6287 (GI*6287*) (*n* = 1) (Table 3). The Tn*5801*-like GI*6287* found in our study, with 99.96% identity to GI*6287*, described previously in *S. pseudintermedius*, has an additional IS*Lmo18* encoding an IS*256* family transposase (Figure 3).

**FIGURE 4:**
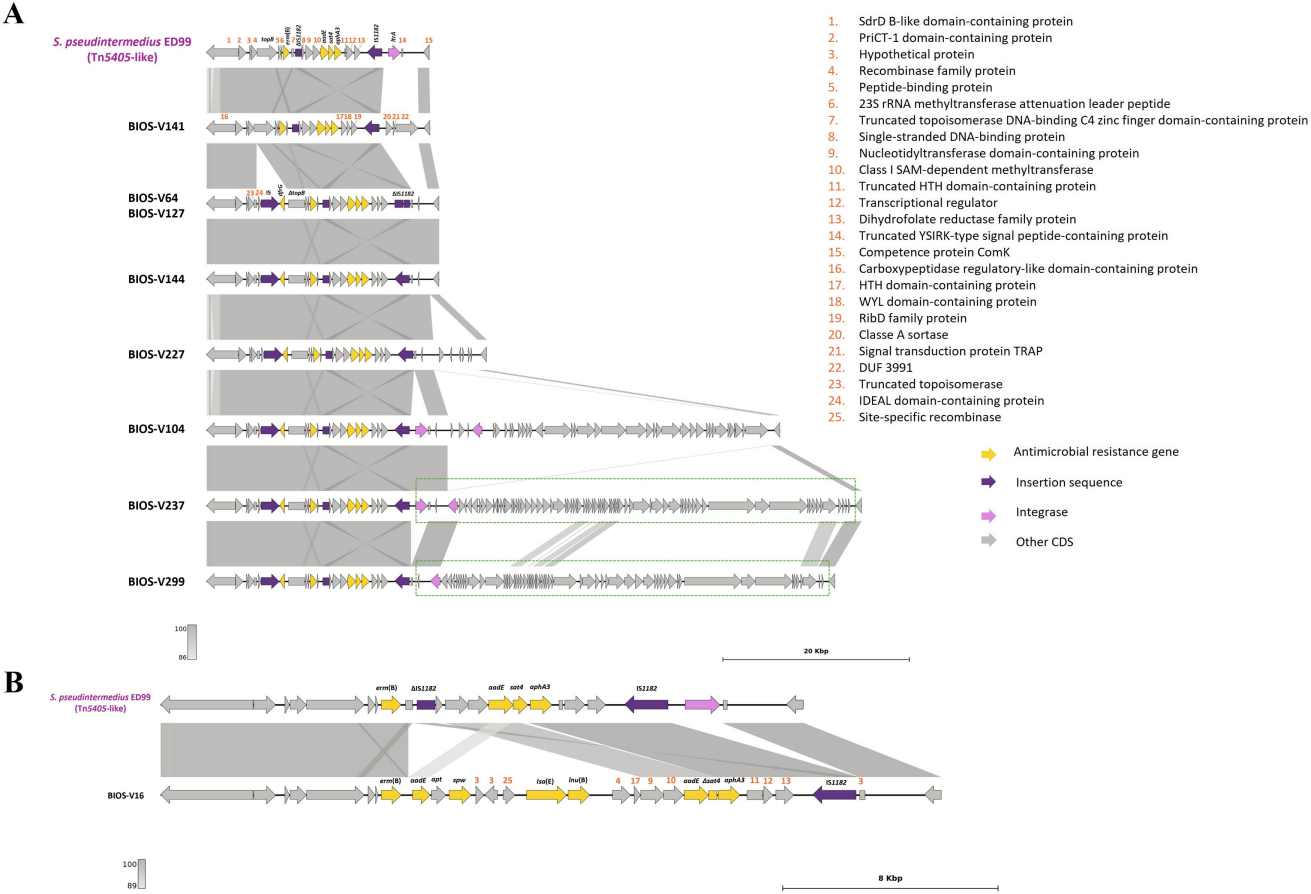
Presentation of Tn*5405*-like and the region until *comK* gene and comparison with strain *S. pseudintermedius* ED99 (NC_017568, [1,833,993:1,857,865]). **(A)** Homology of Tn*5405*-like from ED99 with BIOS-V141, a strain without *dfrG* and seven strains carrying the *dfrG* gene. Phages detected after the transposon are indicated in a dotted green box. **(B)** Representation of BIOS-V16 with the additional four antimicrobial resistance genes [*aadE*, *spw*, *lsa*(E) and *lnu*(B)]. Homology is indicated through a color scale of gray: dark gray (100% homology) to light gray (86% **(A)** or 89% **(B)** homology). Antimicrobial resistance genes are represented in yellow; integrase genes are represented in pink; insertion sequences in purple. Genes colored in gray represent other genes. The figure was generated using Genofig v1.1. The inner lines depict additional regions with homology automatically generated by Genofig.

The genes conferring resistance to aminoglycosides or streptothricin (*aphA3*, *aadE*, *sat4*), macrolide/lincosamide/streptogramin B [*erm*(B)] and/or trimethoprim (*dfrG*) were found in nine strains. These genes were carried by four variants of the Tn*5405*-like element previously identified in *S. pseudintermedius* ED99, three of them differing in the number and functionality of IS*1182* as well as in the presence/absence of *dfrG* that is linked to an additional insertion sequence in the upstream region of *erm*(B) (Figure 4A and Table 3). For two strains (BIOS-V237 and BIOS-V299), an integrase downstream of the Tn*5405*-like element, might have been involved in the insertion of different phages (Figure 4A). The fourth variant carried by BIOS-V16 had a different structure, with a single IS*1182*, a truncated *sat4* gene and four additional genes that confer resistance to aminoglycosides (*aadE*), aminocyclitols (*spw*), pleuromutilins/lincosamides/streptogramin A [*lsa*(E)], and lincosamides [*lnu*(B)] (Figure 4B).

**FIGURE 5:**
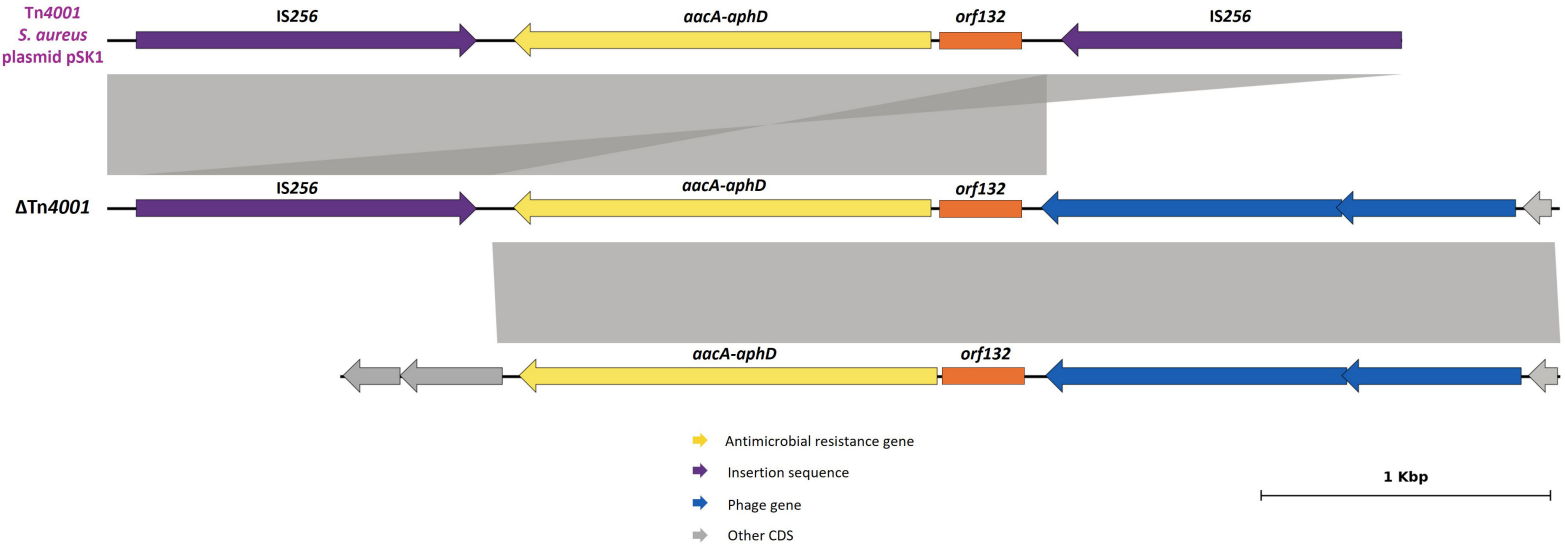
Schematic representations of the truncated Tn*4001* and the region in the vicinity of *aacA-aphD* gene. Comparison with Tn*4001* present in *S. aureus* plasmid pSK1 (accession number: GU565967, [23,577:28,041]). Homology of 100% was detected between sequences. Aminoglycoside resistance gene (*aacA-aphD*) is represented in yellow; *orf132* in orange; insertion sequences in purple; phage-gene related in blue. Genes colored in gray represent other genes. The inner lines depict additional regions with homology automatically generated by Genofig.

The *aacA-aphD* gene, conferring resistance to gentamicin, kanamycin and tobramycin, was located in the chromosomal DNA of ten strains, all with phage-related CDS in its vicinity. For five strains (Table 3), this AMR gene is located on a truncated Tn*4001*, in which the *aacA-aphD* and *orf123* were flanked only by one IS*256* and the surrounding phage-related CDS shared 99.97% identity (50% query cover) with the *S. epidermidis* phage PhiSepi-HH3 (accession no: MT880872.1). In the remaining five strains (BIOS-V16, BIOS-V64, BIOS-V227, BIOS-V262, BIOS-V299) the *aacA-aphD* gene and the *orf123*, were also located in the chromosomal DNA but not flanked by IS*256* (Figure 5).

##### 3.2.2.5 Prophages

Regarding prophage carriage, PHIGARO identified 15 genomes with an average of 136 prophage-like genes per genome (varying from 19 to 345) and two genomes, from ST45 and ST2059 strains, without any prophage-like gene. Forty-six intact, questionable or incomplete prophages were identified, although none harbored AMR genes (Table 3 and Supplementary Table 2). Most prophages were considered intact or incomplete in the different strains. The five ST71 strains analyzed carried 14 out of 27 intact prophages. Four of these five ST71 strains carried *S. pseudintermedius* phage SpST71A (Brooks et al., 2020; Supplementary Table 2). This phage encodes a putative protein of the class B metallo β-lactamase (MBL) superfamily, although no studies have established its β-lactam hydrolytic activity. The gene encoding this putative MBL was also detected in the chromosomal DNA next to *comGA* of the remaining 12 *S. pseudintermedius* strains sequenced, irrespective of beta-lactam resistance phenotype. All ST71 strains harbored one or more phages or phage remnants carrying a gene annotated as *virE*, encoding a putative virulence-associated protein previously detected in *S. aureus* SaPI1 (Lindsay et al., 1998) and in ST71 *S. pseudintermedius* strains (Papić et al., 2021). Several phages found in other lineages also carried the putative virulence gene *virE.* Strains from ST45, ST551, ST1183, ST2059 and ST2061 did not carry intact prophages.

##### 3.2.2.6 Other mobile genetic elements

BIOS-V227 and BIOS-V259 carried the *tet*(K) gene on a plasmid integrated into the chromosomal DNA located between two group II intron reverse transcriptase genes, named pSP-G3C4-like, since it shares identity with pSP-G3C4 described in the *S. pseudintermedius* strain G3C4. This integrated plasmid is closely related to pSP-G3C4 found as free plasmid in the other *tet*(K)-positive strains (BIOS-V104, BIOS-V144 and BIOS-V299). Out of the 12 tetracycline-resistant strains sequenced, BIOS-V227 was the only one with resistance to tetracycline mediated by both *tet*(M) and *tet*(K) (Figure 1).

Variants of the chromosomally integrated 22,000-bp pRE25-like element harboring genes conferring resistance to aminoglycosides and streptothricin (*aphA3*, *aadE*, *sat4*), macrolides/lincosamides/streptogramin B [*erm*(B)], and chloramphenicol (*catA7*) were detected in five strains (Supplementary Figure 1). Four of these variants carried the complete element with four copies of IS*1216* and one IS*1252*, yet lacking IS*256*. One of them also carried an IS*L3* family transposase next to IS*1252*. The fifth variant, present in BIOS-V240, lost an internal 8,103-bp segment, which contained the *erm*(B) and *catA7* genes as well as one IS*1216*.

#### 3.2.3 R-M systems and CRISPR

R-M genes were identified in 15 out of the 17 *S. pseudintermedius* genomes analyzed and were mostly related to R-M Type I (Table 3). These strains contained full intact R-M Type I systems with restriction (HsdR), modification (HsdM) and DNA sequence-recognition (HsdS) subunits. R-M Type II systems with Res and Mod subunits were detected in two MRSP strains, one of which had an element integrated in ΨSCC*mec*_57395_ (BIOS-V292). Strain BIOS-V227 harbored a SCC*mec*V(T)_SL/154_, recently described as carrying a R-M Type III downstream of *orfX* (Duim et al., 2018), which according to REBASE, can be considered an R-M Type IIG.

CRISPR-Cas systems were detected in five of the 17 strains, namely one MRSP (BIOS-V227) and four MSSP (BIOS-V179, BIOS-V212, BIOS-V218, BIOS-V236). The ST551 BIOS-V227 harbored three CRISPR systems; CAS-Type IIC and CAS-Type IIIA, each with the *cas9* and *cas10* signature genes, respectively. The CAS-Type IIIA system was present in two copies, one of them integrated into SSC*mec* V(T)_SL/154_ (Table 3).

## 4 Discussion

Our previous work found a high frequency of resistance to first- and second-line therapeutics recommended for SSTIs caused by *S. pseudintermedius* (Morais et al., 2023) and other staphylococci in companion animals (Costa et al., 2021; Costa et al., 2022; Leal et al., 2023). For *S. aureus*, the literature demonstrates that most resistance genes are found on plasmids or transposons integrated into plasmids, facilitating the transfer of these genes between strains (Malachowa and DeLeo, 2010). The previous data regarding plasmid carriage for *S. pseudintermedius*, initially described as *S. intermedius* (Devriese et al., 2005), indicated a high frequency of plasmid carriage, mostly smaller than 5 kb and associated with resistance to tetracycline and chloramphenicol (Greene and Schwarz, 1992; Schwarz et al., 1995; Schwarz et al., 1998; Werckenthin et al., 2001). The more recent studies, using WGS techniques, confirm the presence of small plasmids in the *S. pseudintermedius* genome, albeit in a variable frequency (Ferrer et al., 2021; Fàbregas et al., 2023; Zehr et al., 2025), and indicate the carriage of *tet*(K) (Viñes et al., 2020; Soimala et al., 2020) but also *qac* genes (Hritcu et al., 2020; Fàbregas et al., 2023) in these plasmids.

Our data suggest that plasmids may not be the most relevant MGE involved in AMR in *S. pseudintermedius*. Despite a high frequency of plasmid carriage among the initial *S. pseudintermedius* collection (31/56, 55.4%), the WGS results indicate that most of the AMR determinants found (*blaZ*, *mecA*, *aacA-aphD*, *aphA3*, *erm*(B), *dfrG*, *tet*(M), *fusC*) were not related to plasmids (Table 5). Indeed, only *tet*(K) was located on plasmid pSP-G3C4, carried by ST71 strains, in accordance with literature (Worthing et al., 2018a; Menandro et al., 2019). pSP-G3C4 and structurally very similar *tet*(K)-carrying plasmids are widespread among various staphylococcal species (Schwarz et al., 1998), including *S. aureus* (Diep et al., 2006), *S. hominis* (Belhout et al., 2023) and *S. epidermidi*s (Zhang et al., 2003). Interestingly, a plasmid with high homology to pSP-G3C4 was also found integrated into the chromosomal DNA of ST551 and ST2109 strains. This pSP-G3C4-like plasmid, located between two group II intron reverse transcriptase genes, is a mobilizable plasmid that carries a *mobV* gene and also the gene *tet*(M) as part of a transposon and was previously found in the chromosomal DNA of ST551 strains (Viñes et al., 2022; Viñes et al., 2024). Detection of the *tet*(K) gene is relevant, as it also confers resistance to doxycycline (Weese et al., 2013), recommended as a second-line therapy for canine pyoderma in dogs (Loeffler et al., 2025). We also detected the efflux gene *qacG*, linked to reduced susceptibility to quaternary ammonium compounds (Costa et al., 2013), located on plasmid pSP5912, previously described in *S. pseudintermedius* (Worthing et al., 2018b). Reduced susceptibility to biocides has been reported in staphylococci that carry *qac* genes (Furi et al., 2013; Costa et al., 2016; Hardy et al., 2018; Worthing et al., 2018b), although the corresponding minimal inhibitory or bactericidal concentrations do not reach the in use recommended concentrations (Couto et al., 2013; Worthing et al., 2018b).

Regarding the MGEs in the chromosomal DNA, SCC*mec* was detected in the 12 MRSP strains. The most frequent type of SCC*mec* was type III, which is usually found in *S. pseudintermedius* ST71 strains (Perreten et al., 2013; Krapf et al., 2019; Wegener et al., 2020), as we observed. SCC*mec* type IVg was found in the ST258 strain and the ΨSCC*mec*_57395_, carrying heavy metal resistance genes, was detected in a ST45 strain, in accordance with other studies (Perreten et al., 2013; Worthing et al., 2018c; Wegener et al., 2020; Bruce et al., 2022). BIOS-V240, from ST2061, previously detected for the first time in our collection, carried SCC*mec*_7017–61515_, a cassette recently described in an ST1200 *S. pseudintermedius* isolated from a dog wound, being the first cassette identified with *mec* gene complex A and a *ccrC1* gene in a non-composite element (MacFadyen and Paterson, 2024). Strain BIOS-V227 (ST551) carries the SCC*mec*V(T)_SL/154_, described by Duim et al. for an ST121 strain (a triple-locus variant of ST551) and harbors a type III R-M systems, a CRISPR/Cas complex and the cadmium resistance gene *cadA* (Duim et al., 2018).

In addition to SCC*mec*, several transposons, integrated into chromosomal DNA, carried AMR determinants. The *blaZ* gene, conferring penicillin resistance, was located either in Tn*552*, Tn*552*-like and/or Tn*553* elements. Tn*552* is frequently detected on *S. aureus* plasmids (Schwarz et al., 2014; Partridge et al., 2018) or integrated into *S. pseudintermedius* chromosomal DNA (McCarthy et al., 2015; Phumthanakorn et al., 2021). Tn*553*, a member of the Tn*554* family, was recently described by Krüger et al. in a porcine MRSA strain and detected *in silico* in MSSP strains (Krüger et al., 2021). In our study, this element was identified in one MRSP and three MSSP strains.

Resistance to tetracycline mediated by *tet*(M) was linked to transposons of the conjugative Tn*916*-like family, namely Tn*916*, Tn*5801* or Tn*5801*-like GI*6287*, all previously reported in *S. pseudintermedius* (McCarthy et al., 2015; de Vries et al., 2016). Genomic islands are relevant for the evolution of bacterial species since they are conserved within strain lineages. The mechanism of mobilization of these elements is not established for *S. pseudintermedius*, but it is expected that horizontal transfer of GI*6287* occurs at a low frequency or under specific conditions, since it lacks the *xis*-like genes necessary for its excision. In *S. aureus*, mobilization of GI*6287* is assisted by temperate helper phages that have specific tail proteins to target the recipient cells that are maintained in related clonal lineages (Moon et al., 2016).

**FIGURE 6:**
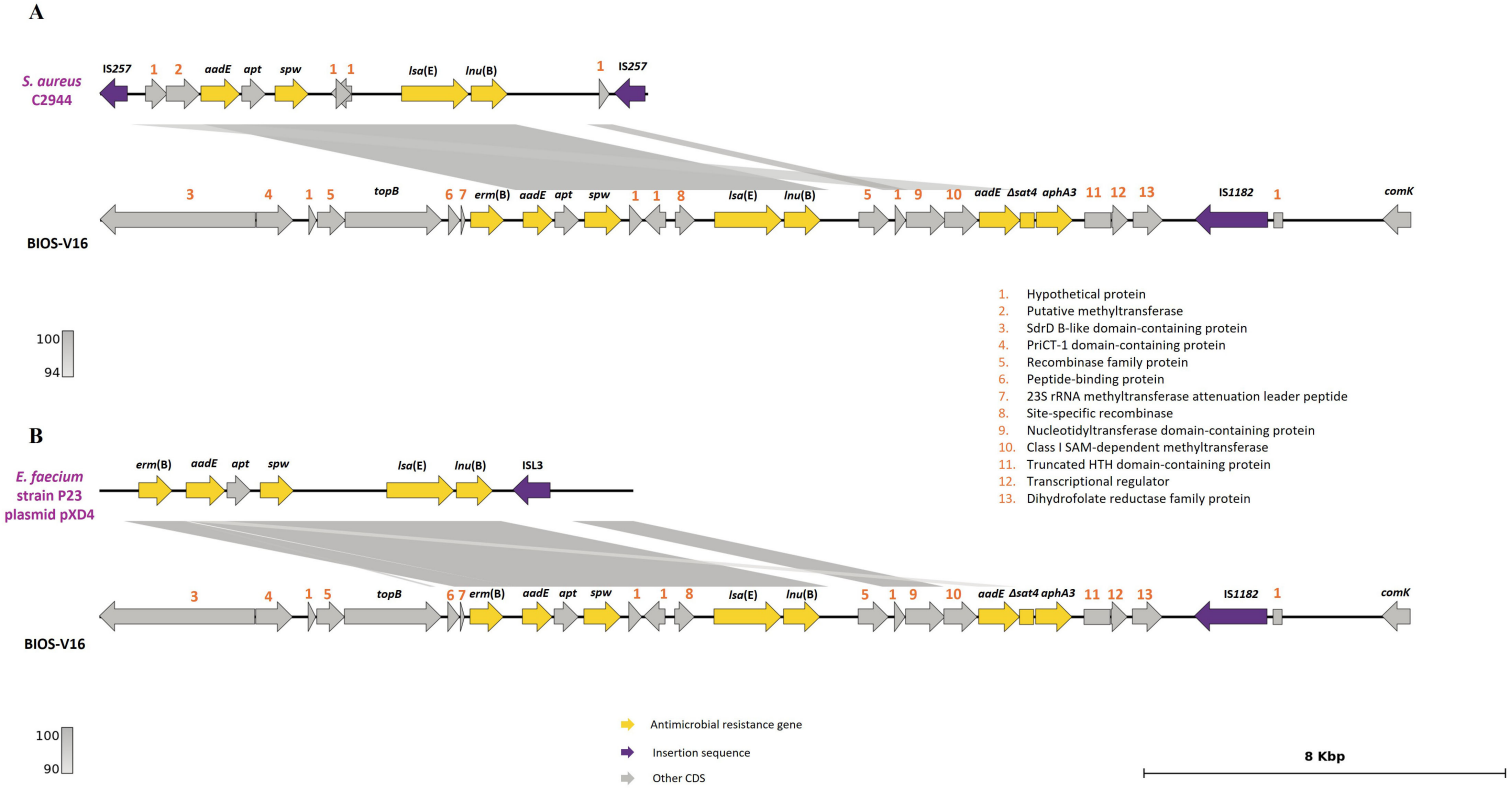
Analysis of the carriage of *erm*(B), *aadE*, *spw*, *lsa*(E) and *lnu*(B) genes by BIOS-V16 strain. **(A)** Comparison with *S. aureus* strain C2944 (JQ861959.1) and **(B)** *Enterococcus faecium* strain P23 plasmid pXD4 (KF421157). Homology is indicated through a color scale of gray: dark gray (100% homology) to light gray (94% **(A)** or 90% **(B)** homology). Antimicrobial resistance genes are represented in yellow; insertion sequence in purple. Genes colored in gray represent other genes. The figure was generated using Genofig v1.1. The inner lines depict additional regions with homology automatically generated by Genofig.

The carriage of Tn*5405*-like variants contributes to AMR, conferring a MDR profile in *S. pseudintermedius* strains (Phumthanakorn et al., 2021). A Tn*5405*-like element was detected in almost all the MRSP strains (9/12), a result in accordance with other reports that verified the predisposition of staphylococci to acquire this transposon following the acquisition of SCC*mec* (McCarthy et al., 2015; Fàbregas et al., 2023). Of interest, the Tn*5405*-like element carried by BIOS-V16 harbored four additional antimicrobial resistance genes (Table 3) in an arrangement previously identified in *S. pseudintermedius* (Viñes et al., 2022). The region encompassing these four genes shares homology with plasmids isolated from a human MRSA (Lozano et al., 2012; Figure 6A) and a porcine *Enterococcus faecium* (Li et al., 2014; Figure 6B).

The *aacA-aphD* gene was detected in a ΔTn*4001* with a single IS*256* integrated in an incomplete phage sequence. Five strains carried a putative Tn*4001*-like variant, not integrated in a phage and lacking IS*256*, suggesting the additional presence of a Tn*4001*-like variant in *S. pseudintermedius*. Several variants of Tn*4001*-like have been reported for *Staphylococcus* spp. (Byrne et al., 1990; Lange et al., 2003; Schwarz et al., 2011; Chanchaithong et al., 2024), mostly caused by the partial deletion of IS*256* or the integration of IS*257*. Zhang et al. identified a Tn*4001* variant without IS*256* on both termini in *Enterococcus faecalis* (Zhang et al., 2018).

Other MGEs identified in our collection included the elements pRE25-like, a new SCC*fus* and prophages.

The pRE25-like element, first described in *Enterococcus* spp. (Werner et al., 2003), is integrated into the *S. pseudintermedius* chromosomal DNA and carries *cat*-*erm*(B)-*aadE*-*sat4*-*aphA3* genes (Kang and Hwang, 2020). We identified variants of this mobile element, one of which carrying only *aadE*-*sat4*-*aphA3*, in ST241 and ST45 strains, in line with other studies (Wegener et al., 2022), but also in the newly described lineages ST2061 and ST2109 (Morais et al., 2023).

Resistance to fusidic acid was detected in five strains, one of them carrying *fusC* in a SCC element described for the first time in *S. pseudintermedius* in this study. This element carries *ccrA4* and *ccrB4* genes and shares similarity with the SCC*mec*-SCC*fus* described earlier in a MRSA strain (Senok et al., 2019). The other four strains resistant to fusidic acid had point mutations resulting in amino acid exchanges in FusA, two of them (FusA:H457Q and FusA:G451V) described previously in *S. aureus* strains with low-level resistance (Besier et al., 2003; O’Neill et al., 2004; Castanheira et al., 2010; Chen et al., 2010) and detected now for the first time in *S. pseudintermedius*. The FusA:I461T alteration was reported previously in *S. pseudintermedius* together with two other mutations and it was related to growth fitness compensation (Frosini et al., 2019; Lim et al., 2020). We also took into consideration the resistance phenotype to rifampicin. The amino acid exchange RpoB:H481N, here reported also for the first time for *S. pseudintermedius*, was earlier described in a MRSA strain of porcine origin, associated with low-level resistance to rifampicin (Li et al., 2016; Schwarz et al., 2018).

The five ST71 strains carried SCC*mec*, Tn*552* and Tn*552*-like as well as Tn*5405*-like variants. Strains belonging to ST258 and ST551, which are considered emerging lineages in the North of Europe, replacing ST71 (Damborg et al., 2016; Kizerwetter-Świda et al., 2017), harbored the same MGEs integrated into the chromosomal DNA, and additionally Tn*553*, Tn*916* and pSP-G3C4-like. Other studies have found different combinations of AMR gene(s)/MGEs/lineages (McCarthy et al., 2015; Phumthanakorn et al., 2021; Fàbregas et al., 2023). ST241, the most frequent ST among the MSSP strains previously studied (Morais et al., 2023) and recently associated to human *S. pseudintermedius* infection (Wegener et al., 2021), showed a MDR profile, conferred by the pRE25-like element. The two MSSP-ST241, MSSP-ST2109, and all MRSP strains studied showed resistance to at least one of the first- and second-line systemic treatment options recommended for SSTIs (clindamycin and cephalosporins). This is highly relevant for the therapy of SSTIs in companion animals since it suggests a possible inefficacy of these antimicrobials as a treatment option, not only for MRSP but also for MSSP strains.

Prophages can carry genes that contribute to AMR, virulence, fitness and adaptation to the host (McCarthy et al., 2015). Intact prophages or prophage-like genes were detected in 88% of the sequenced *S. pseudintermedius* strains and none of them carried AMR determinants, in agreement with previous studies (Wipf et al., 2019; Moodley et al., 2019; Brooks et al., 2020; Phumthanakorn et al., 2021). All ST71 strains except one (BIOS-V299), carried phage SpST71A, described previously in this lineage (Brooks et al., 2020), that disrupts the *comG* operon, a genetic barrier to horizontal gene transfer (HGT). Few data about prophages in *S. pseudintermedius* are available in public databases. BLASTn analysis of our sequences revealed high percentages of identity with several phage sequences but with low query coverage using the NCBI Viruses database (Supplementary Table 2). The BLASTn also allowed the detection of similar prophages in different *S. pseudintermedius* genomes deposited in GenBank, however, these are not identified or classified as phages, hampering prophage identification. Our data suggest that prophages are not related to AMR gene carriage in *S. pseudintermedius*, yet it is known that these MGEs increase strain plasticity, contributing to the genetic diversity of the bacterial population. In addition, they allow a better adaptation of the bacteria to new environments by increasing their pathogenic potential and the transfer of MGEs harboring factors that confer unique virulence characteristics to the bacteria (Naorem et al., 2021; Gummalla et al., 2023).

Restriction-modification and CRISPR/Cas systems are significant genetic barriers that regulate HGT among bacteria, including staphylococci. Four types of R-M systems were reported in *Staphylococcus* species (Sadykov, 2016). In *S. pseudintermedius*, Types I and II are the most frequently described (McCarthy et al., 2015; Brooks et al., 2020; Phumthanakorn et al., 2021). We verified that almost all the strains carried at least one type of R-M system, independently of the number of AMR genes and MGEs carried. R-M type I was the most frequently detected, particularly associated with ST71. A previous study suggested a relation between R-M type and *S. pseudintermedius* clonal lineage (Brooks et al., 2020). That study identified R-M type I in ST71 and ST258, and R-M type II in ST45, in accordance with our findings. Although more studies are needed to confirm the linkage between ST and R-M type observed in *S. pseudintermedius*, data from other bacteria suggest that R-M systems facilitate HGT within the same clonal lineage or between lineages with cognate R-M systems (Oliveira et al., 2016).

The CRISPR/Cas regulatory capacity to control HGT was already described for *Staphylococcus* spp. (Rossi et al., 2017; Mortensen et al., 2021). These systems are not frequently detected in staphylococci of canine origin (Rossi et al., 2019), and only types II and IIIA are known in *S. pseudintermedius* (Brooks et al., 2020; Phumthanakorn et al., 2021). Following previous studies (Brooks et al., 2020; Wegener et al., 2021), these systems were not detected among the ST71 and ST45 strains of our collection. On the other hand, the single ST551 strain carried several CRISPR/Cas systems, also in line with recent reports (Grist et al., 2025), despite its MDR phenotype and multiple MGEs. Since CRISPR/Cas may function as molecular clock, future studies characterizing the spacers from the CRISPR/Cas system could provide information about the origin and the time of these genetic events.

## 5 Conclusion

This study highlights a low diversification of the *S. pseudintermedius* mobilome between clonal lineages. The results obtained in this study indicated that *S. pseudintermedius* has a high proportion of plasmid carriage (> 50%), although with a low diversity and not frequently related to AMR gene carriage. AMR determinants were found mostly within other MGEs integrated into chromosomal DNA, namely Tn*552*, Tn*552*-like, Tn*553*, Tn*5405*-like, Tn*916*, Tn*5801*, Tn*5801*-like GI*6287* and a pRE25-like element. One MSSP strain harbored SCC*fus*, a new element carrying the *fusC* gene, detected, to the best of our knowledge, for the first time in *S. pseudintermedius*. Most of the strains analyzed also carried prophages in their genomes however without AMR genes, suggesting a role in other biological processes such as adaptation to the host and bacterial fitness. The transfer of these MGEs in *S. pseudintermedius* can be controlled by R-M systems, which were present in almost all strains, and CRISPR/Cas systems. The results obtained in this study provide important insights that may lead to a better understanding of MDR in *S. pseudintermedius* towards improved SSTIs treatment in companion animals.

## Data Availability

The datasets presented in this study can be found in online repositories. The names of the repository/repositories and accession number(s) can be found in the article/Supplementary material.

## Footnotes

1
https://www.bioinformatics.babraham.ac.uk/projects/fastqc

2
https://www.genomicepidemiology.org

3
https://cge.food.dtu.dk/services/SCCmecFinder

4
https://tools.neb.com/genomes/

5
https://crisprcas.i2bc.paris-saclay.fr/CrisprCasFinder/Index
