# Supplementary material for: Genomic analysis of the Staphylococcus pseudintermedius mobilome associated with antimicrobial resistance
Source: Front Microbiol. 2025 Oct 8;16:1640322. doi: 10.3389/fmicb.2025.1640322 (PMC12540322; doi:10.3389/fmicb.2025.1640322)
Supplement: Supplementary file 1 [file Table_1.docx]

Supplementary Material

Genomic analysis of the *Staphylococcus pseudintermedius* mobilome associated with antimicrobial resistance

Catarina Morais^1^, Sofia Santos Costa^1^, Dennis Hanke^2,3^, Ana Santos^1^, Henrike Krüger-Haker^2,3^, Constança Pomba^4^, Andrea T. Feßler^2,3^, Stefan Schwarz^2,3^, Isabel Couto^1(*)^

(*) Corresponding author: [icouto@ihmt.unl.pt](mailto:icouto@ihmt.unl.pt)

# Supplementary Figures


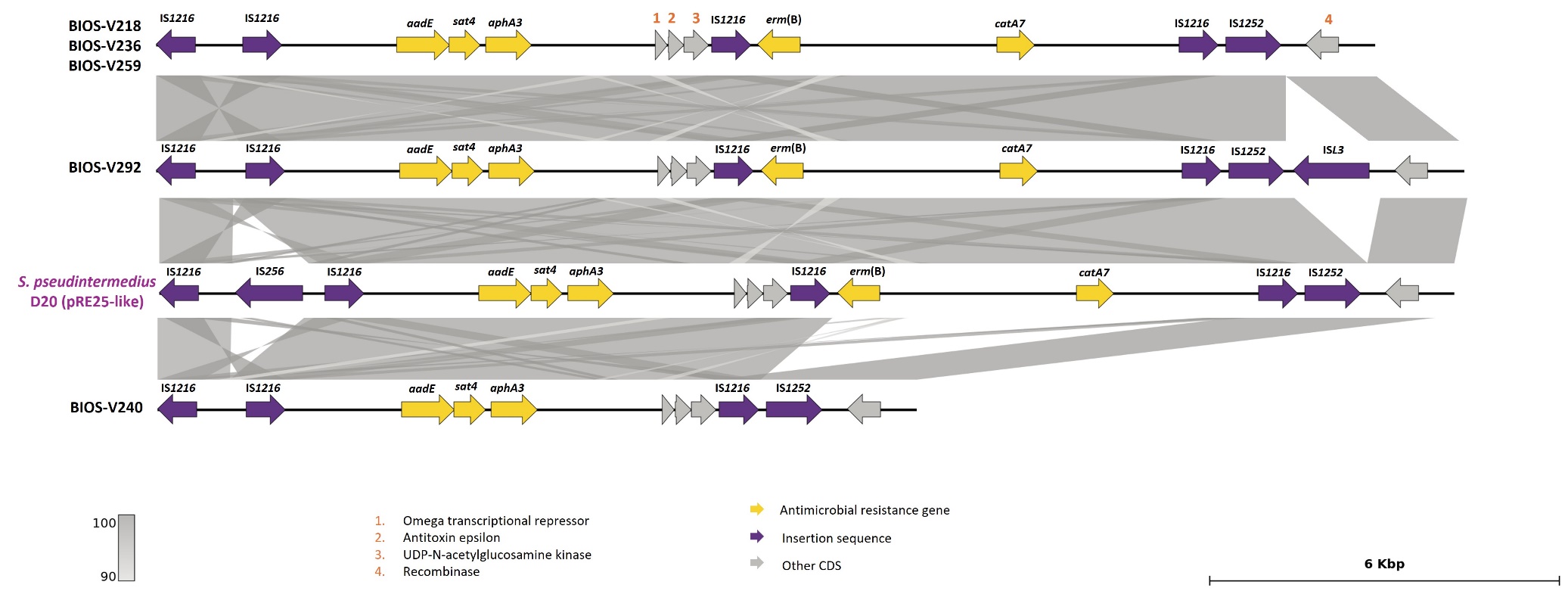


**Supplementary Figure 1** **Presentation of a mobile element isolated from strains BIOS-V218, V236, V259, V292 and V240 and comparison with pRE25-like element from *S. pseudintermedius* strain D20 (MK775653.1).** Homology is indicated through a color scale of grey: dark gray (100% homology) to light gray (90% homology). Antimicrobial resistance genes are represented in yellow; insertion sequences in purple. Genes colored in gray represent other genes. The figure was generated using Genofig vs 1.1. The inner lines depict additional regions with homology automatically generated by Genofig.

# Supplementary Tables

**Supplementary Table 1.** **Resistance properties of the 56 *S. pseudintermedius* selected for plasmid profiling**

| **Strain** | **ST** | **CC** | **Resistance phenotype ^(a)^** | | | | | | | | | | | **Resistance genotype ^(b)^** | | | | | | | | | | | |
| --- | --- | --- | --- | --- | --- | --- | --- | --- | --- | --- | --- | --- | --- | --- | --- | --- | --- | --- | --- | --- | --- | --- | --- | --- | --- |
|  |  |  | **PEN** | **OXA** | **MAC** | **LIN** | **AMG** | **FLQ** | **TET** | **SXT** | **CHL** | **FUS** | **RIF** | ***blaZ*** | ***mecA*** | ***erm*(B)** | ***erm*(C)** | ***aacA-aphD*** | ***aphA3*** | ***tet*(M)** | ***tet*(K)** | ***tet*(L)** | ***cat*** | ***dfrG*** | ***fusC*** |
| BIOS-V7 | 157 | - |  |  |  |  |  |  |  |  |  |  |  |  |  |  |  |  |  |  |  |  |  |  |  |
| BIOS-V25 | 157 | - |  |  |  |  |  |  |  |  |  |  |  |  |  |  |  |  |  |  |  |  |  |  |  |
| BIOS-V26 | 157 | - |  |  |  |  |  |  |  |  |  |  |  |  |  |  |  |  |  |  |  |  |  |  |  |
| **BIOS-V127** | **157** | **-** |  |  |  |  |  |  |  |  |  |  |  |  |  |  |  |  |  |  |  |  |  |  |  |
| BIOS-V224 | 45 | 45 |  |  |  |  |  |  |  |  |  |  |  |  |  |  |  |  |  |  |  |  |  |  |  |
| BIOS-V228 | 45 | 45 |  |  |  |  |  |  |  |  |  |  |  |  |  |  |  |  |  |  |  |  |  |  |  |
| **BIOS-V292** | **45** | **45** |  |  |  |  |  |  |  |  |  |  |  |  |  |  |  |  |  |  |  |  |  |  |  |
| **BIOS-V16** | **265** | **277** |  |  |  |  |  |  |  |  |  |  |  |  |  |  |  |  |  |  |  |  |  |  |  |
| **BIOS-V262** | **118** | **277** |  |  |  |  |  |  |  |  |  |  |  |  |  |  |  |  |  |  |  |  |  |  |  |
| **BIOS-V141** | **258** | **258** |  |  |  |  |  |  |  |  |  |  |  |  |  |  |  |  |  |  |  |  |  |  |  |
| BIOS-V52 | 71 | 71 |  |  |  |  |  |  |  |  |  |  |  |  |  |  |  |  |  |  |  |  |  |  |  |
| **BIOS-V64** | **71** | **71** |  |  |  |  |  |  |  |  |  |  |  |  |  |  |  |  |  |  |  |  |  |  |  |
| BIOS-V99 | 71 | 71 |  |  |  |  |  |  |  |  |  |  |  |  |  |  |  |  |  |  |  |  |  |  |  |
| BIOS-V194 | 71 | 71 |  |  |  |  |  |  |  |  |  |  |  |  |  |  |  |  |  |  |  |  |  |  |  |
| BIOS-V223 | 71 | 71 |  |  |  |  |  |  |  |  |  |  |  |  |  |  |  |  |  |  |  |  |  |  |  |
| BIOS-V285 | 71 | 71 |  |  |  |  |  |  |  |  |  |  |  |  |  |  |  |  |  |  |  |  |  |  |  |
| BIOS-V302 | 71 | 71 |  |  |  |  |  |  |  |  |  |  |  |  |  |  |  |  |  |  |  |  |  |  |  |
| **BIOS-V237** | **71** | **71** |  |  |  |  |  |  |  |  |  |  |  |  |  |  |  |  |  |  |  |  |  |  |  |
| BIOS-V40 | 71 | 71 |  |  |  |  |  |  |  |  |  |  |  |  |  |  |  |  |  |  |  |  |  |  |  |
| BIOS-V97 | 71 | 71 |  |  |  |  |  |  |  |  |  |  |  |  |  |  |  |  |  |  |  |  |  |  |  |
| **BIOS-V104** | **71** | **71** |  |  |  |  |  |  |  |  |  |  |  |  |  |  |  |  |  |  |  |  |  |  |  |
| BIOS-V143 | 71 | 71 |  |  |  |  |  |  |  |  |  |  |  |  |  |  |  |  |  |  |  |  |  |  |  |
| BIOS-V53 | 71 | 71 |  |  |  |  |  |  |  |  |  |  |  |  |  |  |  |  |  |  |  |  |  |  |  |
| BIOS-V83 | 71 | 71 |  |  |  |  |  |  |  |  |  |  |  |  |  |  |  |  |  |  |  |  |  |  |  |
| BIOS-V108 | 71 | 71 |  |  |  |  |  |  |  |  |  |  |  |  |  |  |  |  |  |  |  |  |  |  |  |
| BIOS-V125 | 71 | 71 |  |  |  |  |  |  |  |  |  |  |  |  |  |  |  |  |  |  |  |  |  |  |  |
| BIOS-V131 | 71 | 71 |  |  |  |  |  |  |  |  |  |  |  |  |  |  |  |  |  |  |  |  |  |  |  |
| **BIOS-V144** | **71** | **71** |  |  |  |  |  |  |  |  |  |  |  |  |  |  |  |  |  |  |  |  |  |  |  |
| BIOS-V146 | 71 | 71 |  |  |  |  |  |  |  |  |  |  |  |  |  |  |  |  |  |  |  |  |  |  |  |
| BIOS-V207 | 71 | 71 |  |  |  |  |  |  |  |  |  |  |  |  |  |  |  |  |  |  |  |  |  |  |  |
| BIOS-V264 | 71 | 71 |  |  |  |  |  |  |  |  |  |  |  |  |  |  |  |  |  |  |  |  |  |  |  |
| BIOS-V280 | 71 | 71 |  |  |  |  |  |  |  |  |  |  |  |  |  |  |  |  |  |  |  |  |  |  |  |
| BIOS-V286 | 71 | 71 |  |  |  |  |  |  |  |  |  |  |  |  |  |  |  |  |  |  |  |  |  |  |  |
| **BIOS-V299** | **71** | **71** |  |  |  |  |  |  |  |  |  |  |  |  |  |  |  |  |  |  |  |  |  |  |  |
| **BIOS-V227** | **551** | **551** |  |  |  |  |  |  |  |  |  |  |  |  |  |  |  |  |  |  |  |  |  |  |  |
| BIOS-V270 | 25 | 25 |  |  |  |  |  |  |  |  |  |  |  |  |  |  |  |  |  |  |  |  |  |  |  |
| BIOS-V213 | 422 | 422 |  |  |  |  |  |  |  |  |  |  |  |  |  |  |  |  |  |  |  |  |  |  |  |
| BIOS-V276 | 497 | 309 |  |  |  |  |  |  |  |  |  |  |  |  |  |  |  |  |  |  |  |  |  |  |  |
| BIOS-V233 | 924 | - |  |  |  |  |  |  |  |  |  |  |  |  |  |  |  |  |  |  |  |  |  |  |  |
| **BIOS-V240** | **2061** | **-** |  |  |  |  |  |  |  |  |  |  |  |  |  |  |  |  |  |  |  |  |  |  |  |
| BIOS-V241 | 2061 | - |  |  |  |  |  |  |  |  |  |  |  |  |  |  |  |  |  |  |  |  |  |  |  |
| BIOS-V105 | 2056 | 2166 |  |  |  |  |  |  |  |  |  |  |  |  |  |  |  |  |  |  |  |  |  |  |  |
| BIOS-V119 | 2057 | - |  |  |  |  |  |  |  |  |  |  |  |  |  |  |  |  |  |  |  |  |  |  |  |
| BIOS-V217 | 2060 | - |  |  |  |  |  |  |  |  |  |  |  |  |  |  |  |  |  |  |  |  |  |  |  |
| BIOS-V140 | 241 | 241 |  |  |  |  |  |  |  |  |  |  |  |  |  |  |  |  |  |  |  |  |  |  |  |
| **BIOS-V218** | **241** | **241** |  |  |  |  |  |  |  |  |  |  |  |  |  |  |  |  |  |  |  |  |  |  |  |
| **BIOS-V236** | **241** | **241** |  |  |  |  |  |  |  |  |  |  |  |  |  |  |  |  |  |  |  |  |  |  |  |
| **BIOS-V179** | **2059** | **-** |  |  |  |  |  |  |  |  |  |  |  |  |  |  |  |  |  |  |  |  |  |  |  |
| BIOS-V84 | 2099 | - |  |  |  |  |  |  |  |  |  |  |  |  |  |  |  |  |  |  |  |  |  |  |  |
| BIOS-V101 | 2102 | - |  |  |  |  |  |  |  |  |  |  |  |  |  |  |  |  |  |  |  |  |  |  |  |
| **BIOS-V259** | **2109** | **-** |  |  |  |  |  |  |  |  |  |  |  |  |  |  |  |  |  |  |  |  |  |  |  |
| **BIOS-V212** | **1183** | **-** |  |  |  |  |  |  |  |  |  |  |  |  |  |  |  |  |  |  |  |  |  |  |  |
| BIOS-V29 | 2055 | - |  |  |  |  |  |  |  |  |  |  |  |  |  |  |  |  |  |  |  |  |  |  |  |
| BIOS-V103 | n.d. | - |  |  |  |  |  |  |  |  |  |  |  |  |  |  |  |  |  |  |  |  |  |  |  |
| BIOS-V37 | n.d. | - |  |  |  |  |  |  |  |  |  |  |  |  |  |  |  |  |  |  |  |  |  |  |  |
| BIOS-V145 | n.d. | - |  |  |  |  |  |  |  |  |  |  |  |  |  |  |  |  |  |  |  |  |  |  |  |

Data from Morais *et al*., 2023, updated according to WGS results.

(a) resistance/intermediate (dark blue)/susceptible (white); (b) acquired resistance gene (orange)/absent (white); ST: sequence type; CC: clonal complex; PEN: penicillin; OXA: oxacillin; MAC: macrolides; LIN: lincosamides; AMG: aminoglycosides; FLQ: fluoroquinolones; TET: tetracyclines; SXT: trimethoprim-sulfamethoxazole; CHL: chloramphenicol; FUS: fusidic acid; RIF: rifampicin; n.d.: not determined. The strains in bold correspond to the 17 selected for WGS analysis.

**Supplementary Table 2.** **BlastN results obtained against the viruses GenBank database for the prophage sequences identified by PHASTEST.**

| **ST** | **Strain** | **Length (kb)** | **Completeness ^(1)^**  **(PHASTEST)** | **BLAST result (best hit)** |  |  |
| --- | --- | --- | --- | --- | --- | --- |
|  |  |  |  | **Description** | **% Identity** | **% Query cover** |
| **71** | **BIOS-V64** | 45.8 | Intact | *Staphylococcus* phage phiSP38-1 | 98.96 | 16.0 |
|  |  | 48.3 | Intact | *Staphylococcus* phage phiSP119-1 | 97.25 | 75.0 |
|  |  | 37.5 | Questionable | Unnamed 1 (*Caudoviricetes* sp.) | 78.82 | 5.0 |
|  |  | 51.6 | Incomplete | *Staphylococcus* phage phiSP119-2 | 99.99 | 97.0 |
|  |  | 44.3 | --^(2)^ | *Staphylococcus* phage SpST71A | 99.9 | 100 |
|  | **BIOS-V104** | 45.8 | Intact | *Staphylococcus* phage phiSP38-1 | 99.39 | 11.0 |
|  |  | 59.0 | Intact | *Staphylococcus* phage SN13 | 99.20 | 6.0 |
|  |  | 79.6 | Intact | *Staphylococcus* phage phiSP119-3 | 89.03 | 10.0 |
|  |  | 52.9 | Intact | *Staphylococcus* phage phiSP119-2 | 97.01 | 63.0 |
|  |  | 54.1 | Intact | *Staphylococcus* phage phiSP119-1 | 96.17 | 65.0 |
|  |  | 44.3 | --^(2)^ | *Staphylococcus* phage SpST71A | 99.9 | 100 |
|  | **BIOS-V144** | 45.8 | Intact | *Staphylococcus* phage phiSP38-1 | 99.32 | 11.0 |
|  |  | 51.6 | Incomplete | *Staphylococcus* phage phiSP119-2 | 97.01 | 64.0 |
|  |  | 27.0 | Incomplete | *Staphylococcus* phage phiSP119-3 | 93.79 | 1.0 |
|  |  | 44.3 | --^(2)^ | *Staphylococcus* phage SpST71A | 99.9 | 100 |
|  | **BIOS-V237** | 43.8 | Intact | *Staphylococcus* phage SpT252 | 99.95 | 82.0 |
|  |  | 46.8 | Intact | *Staphylococcus* phage SN11 | 99.27 | 26.0 |
|  |  | 78.3 | Intact | *Staphylococcus* phage phiSP119-3 | 89.03 | 10.0 |
|  |  | 51.6 | Incomplete | *Staphylococcus* phage phiSP119-2 | 97.00 | 64.0 |
|  |  | 53.5 | Incomplete | *Staphylococcus* phage phiSP15-1 | 88.01 | 48.0 |
|  |  | 42.8 | Incomplete | *Staphylococcus* phage SP197 DNA | 98.29 | 84.0 |
|  |  | 57.3 | Incomplete | *Staphylococcus* phage phiSP38-1 | 99.39 | 9.0 |
|  |  | 44.3 | --^(2)^ | *Staphylococcus* phage SpST71A | 99.9 | 100 |
|  | **BIOS-V299** | 45.8 | Intact | *Staphylococcus* phage phiSP38-1 | 99.39 | 11.0 |
|  |  | 58.4 | Intact | *Staphylococcus* phage SP197 DNA | 98.80 | 59.0 |
|  |  | 46.2 | Intact | *Staphylococcus* phage phiSP119-1 | 97.34 | 82.0 |
|  |  | 37.5 | Questionable | Unnamed 1 (*Caudoviricetes* sp.) | 78.82 | 5.0 |
|  |  | 52.0 | Incomplete | *Staphylococcus* phage phiSP15-1 | 88.01 | 51.0 |
|  |  | 46.6 | Incomplete | *Staphylococcus* phage SN11 | 99.49 | 34.0 |
| **258** | **BIOS-V141** | 45.3 | Intact | *Staphylococcus* phage SN10 | 93.47 | 18.0 |
|  |  | 46.6 | Incomplete | *Staphylococcus* phage phiSP119-2 | 98.51 | 14.0 |
|  |  | 44.8 | Incomplete | *Staphylococcus* phage SN10 | 95.53 | 70.0 |

**Supplementary Table 2. *(Continuation).***

| **ST** | **Strain** | | **Length (kb)** | **Completeness^1^**  **(PHATEST)** | | | **BLAST result (best hit)** |  | |  | |
| --- | --- | --- | --- | --- | --- | --- | --- | --- | --- | --- | --- |
|  |  |  |  |  |  |  | **Description** | **% Identity** | | **% Query cover** | |
| **118** | | **BIOS-V262** | 45.9 | | Intact | *Staphylococcus* phage SP197 DNA | | | 97.28 | | 71.0 |
|  |  |  | 46.6 | | Incomplete | *Staphylococcus* phage phiSP119-2 | | | 98.51 | | 14.0 |
| **265** | | **BIOS-V16** | 53.2 | | Intact | *Staphylococcus* phage SP276 DNA | | | 94.37 | | 17.0 |
|  |  |  | 57.9 | | Intact | *Staphylococcus* phage SP197 DNA | | | 97.29 | | 60.0 |
|  |  |  | 46.6 | | Intact | *Staphylococcus* phage phiSP119-2 | | | 98.51 | | 14.0 |
| **551** | | **BIOS-V227** | 63.2 | | Incomplete | *Staphylococcus* phage SpT99F3 | | | 99.56 | | 24.0 |
| **157** | | **BIOS-V127** | 48.2 | | Intact | *Staphylococcus* phage phiSP119-2 | | | 97.64 | | 58.0 |
| **2061** | | **BIOS-V240** | 45.1 | | Incomplete | *Staphylococcus* phage SpT99F3 | | | 92.72 | | 11.0 |
| **241** | | **BIOS-V218** | 46.6 | | Intact | Unnamed 1 (*Caudoviricetes* sp.) | | | 77.85 | | 14.0 |
|  |  |  | 57.2 | | Intact | *Staphylococcus* phage phiSP15-1 | | | 97.09 | | 31.0 |
|  |  |  | 44.5 | | Incomplete | *Staphylococcus* phage phiSP119-2 | | | 93.98 | | 32.0 |
|  |  | **BIOS-V236** | 52.8 | | Intact | Unnamed 2 (*Caudoviricetes* sp.) | | | 99.31 | | 74.0 |
|  |  |  | 44.5 | | Intact | *Staphylococcus* phage phiSP119-2 | | | 93.97 | | 32.0 |
| **1183** | | **BIOS-V212** | 36.2 | | Incomplete | Unnamed 1 (*Caudoviricetes* sp.) | | | 77.81 | | 18.0 |
| **2109** | | **BIOS-V259** | 50.0 | | Intact | Unnamed 2 (*Caudoviricetes* sp.) | | | 98.94 | | 77.0 |
|  |  |  | 22.5 | | Incomplete | Unnamed 3 (*Caudoviricetes* sp.) | | | 99.6 | | 64.0 |
|  |  |  | 55.8 | | Intact | *Staphylococcus* phage phiSP119-2 | | | 93.98 | | 25.0 |
|  |  |  | 58.6 | | Intact | *Staphylococcus* phage SN13 | | | 97.98 | | 54 |

ST: sequence-type

1 - PHATEST score: intact < 90, questionable 70-90, incomplete < 70.

2 - Prophage detected with Geneious by comparison with data from Brooks *et al.*, 2020.

Each color represents a unique phage.

**Accession numbers**: *Staphylococcus* phage phiSP38-1 (MK075002.1); *Staphylococcus* phage phiSP119-1 (MK075004.1); Unnamed 1 (*Caudoviricetes* sp.) (BK031568.1); Unnamed 2 (*Caudoviricetes* sp.) (OR221507.1); Unnamed 3 (*Caudoviricetes* sp.) (OR222831.1); *Staphylococcus* phage phiSP119-2 (MK075005.1); *Staphylococcus pseudintermedius* strain 081661 (CP016073, *Staphylococcus* phage SpST71A [1318351:1370836]); *Staphylococcus* phage SN13 (MF428478); *Staphylococcus* phage phiSP119-3 (MK075006.1); *Staphylococcus* phage SpT252 (KX827370.1); *Staphylococcus* phage SN11 (MF428479.1); *Staphylococcus* phage phiSP15-1 (MK075001.1); *Staphylococcus* phage SP197 DNA (NC_055022.1); *Staphylococcus* phage SN10 (MF428480); *Staphylococcus* phage SP276 DNA (NC_055023.1); *Staphylococcus* phage SpT99F3 (KX827371.1).
